# m^6^A regulators as predictive biomarkers for chemotherapy benefit and potential therapeutic targets for overcoming chemotherapy resistance in small-cell lung cancer

**DOI:** 10.1186/s13045-021-01173-4

**Published:** 2021-11-10

**Authors:** Zhihui Zhang, Chaoqi Zhang, Zhaoyang Yang, Guochao Zhang, Peng Wu, Yuejun Luo, Qingpeng Zeng, Lide Wang, Qi Xue, Yi Zhang, Nan Sun, Jie He

**Affiliations:** 1 Department of Thoracic Surgery, National Cancer Center/National Clinical Research Center for Cancer/Cancer Hospital, Chinese Academy of Medical Sciences and Peking Union Medical College, Beijing, 100021 China; 2 Department of Pathology, National Cancer Center/National Clinical Research Center for Cancer/Cancer Hospital, Chinese Academy of Medical Sciences and Peking Union Medical College, Beijing, 100021 China; 3 Biotherapy Center, The First Affiliated Hospital of Zhengzhou University, Zhengzhou, 450052 Henan China

**Keywords:** Small-cell lung cancer, m^6^A regulators, Epigenetic modification, Chemotherapy resistance, Individualized medicine

## Abstract

Small-cell lung cancer (SCLC) is a devastating subtype of lung cancer with few therapeutic options. Despite the advent of immunotherapy, platinum-based chemotherapy is still the irreplaceable first-line therapy for SCLCs. However, drug resistance will invariably occur in most patients and the outcomes are heterogeneous. Therefore, clinically feasible classification strategies and potential therapeutic targets for overcoming chemotherapy resistance are urgently needed. *N*^6^-methyladenosine (m^6^A) is a novel epigenetic decisive factor that is involved in tumor progression and drug resistance. However, almost nothing is known about m^6^A modification in SCLC. Here, we assessed 200 SCLC samples from patients who underwent chemotherapy from three different cohorts, including a validation cohort containing 71 cases with qPCR data and an independent cohort containing 79 cases with immunohistochemistry data (quantified as H-score). We systematically characterized the predictive landscape of m^6^A regulators in SCLC patients following with chemotherapy. Using the LASSO Cox model, we built a seven-regulator-based (*ZCCHC4*, *IGF2BP3*, *ALKBH5*, *YTHDF3*, *METTL5*, *G3BP1*, and *RBMX*) chemotherapy benefit predictive classifier (m^6^A score) and subsequently validated the classifier in two other cohorts. Time-dependent ROC and C-index analyses showed that the m^6^A score to possessed superior predictive power for chemotherapy benefit in comparison with other clinicopathological parameters. A multicohort multivariate analysis revealed that the m^6^A score is an independent factor that affects survival benefit across multiple cohorts. Our in vitro experimental results revealed that three regulators—ZCCHC4, G3BP1, and RBMX—may serve as promising novel therapeutic targets for overcoming chemoresistance in SCLCs. Our results, for the first time, demonstrate the predictive significance of m^6^A regulators for chemotherapy benefit, as well as their potential as therapeutic targets for overcoming chemotherapy resistance in SCLC patients. The m^6^A score was found to be a reliable prognostic tool that may help guide chemotherapy decisions for patients with SCLC.

**Supplementary Information:**

The online version contains supplementary material available at 10.1186/s13045-021-01173-4.

## To the editor

Lung cancer remains the leading cause of cancer-associated mortality [1, 2], while small-cell lung cancer (SCLC) comprises approximately 15% of these cases [3]. SCLC is characterized by poor prognosis with a 5-year survival rate of less than 7% [4]. Despite the advent of immunotherapies, the prognosis for SCLCs remains grim [5]. Chemotherapy is still the irreplaceable first-line therapy [6]. However, drug resistance occurs in most patients [4]. *N*^6^-methyladenosine (m^6^A) is a new epigenetic decisive factor that is related to chemotherapy resistance [7], via a mechanism that involves several regulators [8]. Although multiple epigenetic modifications have been tightly linked to drug resistance in SCLC [9], the role of the m^6^A modification in SCLC remains elusive.

**Fig. 1:**
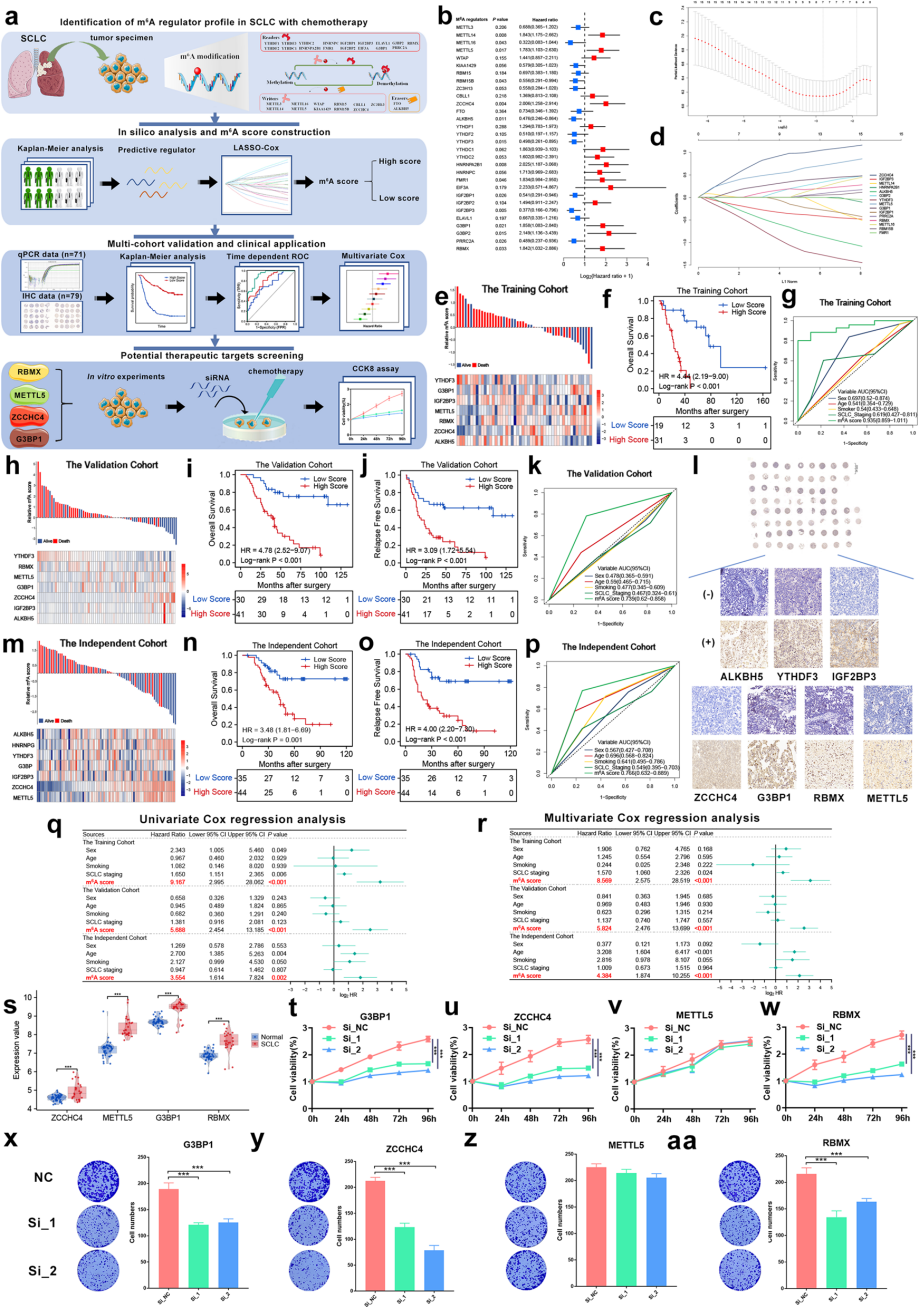
Identification of m^6^A regulators as predictive biomarkers and potential therapeutic targets in small-cell lung cancer with chemotherapy. **a** The work flow of this study. Thirty m^6^A regulators were selected from several recently published studies. The predictive regulators were filtered out through Kaplan–Meier curve analysis. The m^6^A score was constructed using the LASSO Cox regression model and the training cohort. The m^6^A score was validated in two different cohorts with qPCR data and immunohistochemistry data. Finally, the therapeutic potential of several regulators was explored through in vitro experiments. **b** A forest plot of the optimum cutoff survival analysis of the m^6^A regulators in SCLC patients from the training cohort who underwent chemotherapy. **c** The LASSO model was selected to determine the partial likelihood deviance of different numbers of variables, and 100-fold cross-validation was chosen. **d** Distribution of the LASSO coefficients of 15 significant regulators. **e** Distribution of the seven regulators comprising the m^6^A score, the corresponding m^6^A score, and survival status in the training cohort. **f** Survival curve of OS for patients from the training cohort. **g** Time-dependent ROC curves comparing the prognostic accuracy of the m^6^A score with other clinicopathological parameters at 5 years in the training cohort. **h** Distribution of the seven regulators comprising the m^6^A score, the corresponding m^6^A score, and survival status in the validation cohort with qPCR data. **i** Survival curve of OS for patients from the validation cohort. **j** Survival curve of RFS for patients from the validation cohort. **k** Time-dependent ROC curves comparing the prognostic accuracy of the m^6^A score with other clinicopathological parameters at 5 years in the validation cohort. **l** Representative immunohistochemistry images of the seven regulators comprising the m^6^A score from the tissue microarray; (+) high expression, (−) low expression (40 ×). **m** Distribution of the seven regulators comprising the m^6^A score, the corresponding m^6^A score, and survival status in the independent cohort. **n** Survival curve of OS for patients from the independent cohort. **o** Survival curve of RFS for patients from the independent cohort. **p** Time-dependent ROC curves comparing the prognostic accuracy of the m^6^A score with other clinicopathological parameters at 5 years in the independent cohort. **q** Univariate Cox regression analysis of clinicopathological factors and the m^6^A score for OS in patients across multiple cohorts. **r** Multivariate Cox regression analysis of clinicopathological factors and the m^6^A score for OS in patients across multiple cohorts. **s** Distribution of the four selected regulators in normal lung and SCLC tissues from GSE40275. **t**, **x** Knocking down the expression of G3BP1 significantly increased the sensitivity of SCLC cells to cisplatin. **u**, **y** Knocking down the expression of ZCCH4 significantly increased the sensitivity of SCLC cells to cisplatin. **v**, **z** Knocking down the expression of METTL5 had no effect on the sensitivity of SCLC cells to cisplatin. **w**, **aa** Knocking down the expression of RBMX significantly increased the sensitivity of SCLC cells to cisplatin

The workflow is displayed in Fig. 1a. In total, 30 regulators were assessed (Additional file 1: Table S1). We determined the survival benefit predictive values of these regulators in patients who underwent adjuvant chemotherapy (ACT). Half of the regulators (15/30) exhibited significant clinical relevance (Fig. 1b, Additional file 2: Fig. S1, Additional file 1: Table S2), indicating that m^6^A modification may contribute to chemotherapy efficacy. We hypothesized that an m^6^A regulator-based signature might predict the benefit of ACT. We obtained data for 200 SCLC samples from three cohorts of patients who underwent ACT to construct the m^6^A score (Additional file 1: Table S3). Considering the collinearity of the data (Additional file 2: Fig. S2), the LASSO Cox model was selected. Seven regulators (*ZCCHC4*, *IGF2BP3*, *ALKBH5*, *YTHDF3*, *METTL5*, *G3BP1*, and *RBMX*) were identified (Fig. 1c, d) and used to generate m^6^A scores (Fig. 1e, Additional file 3). Patients with high m^6^A scores had significantly worse overall survival (OS) (Fig. 1f, *P* < 0.001). The m^6^A score showed excellent performance across different years (Additional file 2: Fig. S3a) and better accuracy than other clinicopathological parameters for predicting the OS benefit (Fig. 1g, Additional file 2: Fig. S3b).

A set of 70 surgical specimens from patients who underwent ACT (qPCR data) was selected as a validation cohort (Fig. 1h, Additional file 1: Table S4). Similarly, high-score patients had shorter OS (Fig. 1i, *P* < 0.001) and relapse-free survival (RFS) (Fig. 1j, *P* < 0.001). The m^6^A score also performed well at different follow-up times (Additional file 2: Fig. S4a, b) and had an AUC (0.739) and C-index (0.833) that were higher than those of other parameters (Fig. 1k, Additional file 2: Fig. S4c). The m^6^A score also showed superiority for predicting the RFS benefit (Additional file 2: Fig. S4d, e). Furthermore, the m^6^A score was also calculated based on the H-scores in an independent cohort containing immunohistochemistry data (*n* = 79) (Fig. 1l, m). Low-score patients exhibited longer OS (Fig. 1n, *P* = 0.001) and RFS (Fig. 1o, *P* < 0.001). The AUCs for OS and RFS (Additional file 2: Fig. S5a, b) indicated that the protein level-based classifier is also a stable predictor. The m^6^A score also achieved a high AUC of 0.766 (Fig. 1p) and a C-index of 0.756 (Additional file 2: Fig. S5c). The advantage of the m^6^A score was also confirmed in predicting the RFS (Additional file 2: Fig. S5d, e). Additionally, the m^6^A score was the only stable and independent factor affecting survival across multiple cohorts (Fig. 1q, r, Additional file 2: Fig. S6, *P* < 0.001). As far as we know, the m^6^A score is the first molecular model using a large cohort of SCLCs who underwent chemotherapy. Large-scale analyses of SCLC samples are extremely rare because of the difficulties associated with obtaining tumor specimens within standard clinical settings.

Given that four of the regulators—*ZCCHC4*, *METTL5*, *G3BP1*, and *RBMX*—that comprised the m^6^A score were risk factors (coefficient > 0), we investigated their therapeutic potential for overcoming chemotherapy resistance. All four of these regulators were dramatically up-regulated in the SCLC samples (Fig. 1s). In vitro experiments found that knocking down the expression of *ZCCHC4*, *G3BP1*, or *RBMX* significantly increased the sensitivity of SCLC cells to chemotherapeutic drugs. In contrast, down-regulation of METTL5 had no notable influence on drug sensitivity (Fig. 1t–w, x–aa, Additional file 2: Fig. S7). These results suggest that m^6^A regulators are promising novel therapeutic targets for overcoming chemoresistance.

For the first time, we found that m^6^A regulators can predict chemotherapy benefit, potentially minimizing the risk of chemotherapy resistance in SCLCs. The m^6^A score is a reliable prognostic tool for guiding chemotherapy options. Patients with low m^6^A scores are more likely to benefit from ACT. In contrast, patients with high m^6^A scores would likely be better served by utilizing other treatment regimens or participating in clinical trials with new strategies, rather than enduring the toxic side effects of chemotherapy that might provide no therapeutic benefit. This in vitro study also indicated that therapeutic interventions targeting m^6^A regulators may provide patients with enhanced and durable responses.

## Supplementary Information

**Additional file 1.** Supplementary Tables.

**Additional file 2.** Supplementary Figures.

**Additional file 3.** Supplementary materials and methods.

## Data Availability

All data that support the findings of this study are available to researchers on reasonable request.

## Abbreviations

SCLC: Small-cell lung cancer
m^6^A: *N*^6^-Methyladenosine
ACT: Adjuvant chemotherapy
LASSO: Least absolute shrinkage and selection operator
OS: Overall survival
AUC: Area under the curve
RFS: Relapse-free survival

## References

[1] Sung H, Ferlay J, Siegel RL, Laversanne M, Soerjomataram I, Jemal A (2021). Global Cancer Statistics 2020: GLOBOCAN estimates of incidence and mortality Worldwide for 36 cancers in 185 countries. CA Cancer J Clin.

[2] Majeed U, Manochakian R, Zhao Y, Lou Y (2021). Targeted therapy in advanced non-small cell lung cancer: current advances and future trends. J Hematol Oncol.

[3] Owen DH, Giffin MJ, Bailis JM, Smit MD, Carbone DP, He K (2019). DLL3: an emerging target in small cell lung cancer. J Hematol Oncol.

[4] Rudin CM, Brambilla E, Faivre-Finn C, Sage J (2021). Small-cell lung cancer. Nat Rev Dis Primers.

[5] Iams WT, Porter J, Horn L (2020). Immunotherapeutic approaches for small-cell lung cancer. Nat Rev Clin Oncol.

[6] Yang S, Zhang Z, Wang Q (2019). Emerging therapies for small cell lung cancer. J Hematol Oncol.

[7] Huang H, Weng H, Chen J (2020). m(6)A modification in coding and non-coding RNAs: roles and therapeutic implications in cancer. Cancer Cell.

[8] Zhao Y, Shi Y, Shen H, Xie W (2020). m(6)A-binding proteins: the emerging crucial performers in epigenetics. J Hematol Oncol.

[9] Khan P, Siddiqui JA, Maurya SK, Lakshmanan I, Jain M, Ganti AK (2020). Epigenetic landscape of small cell lung cancer: small image of a giant recalcitrant disease. Semin Cancer Biol..

