# Supplementary material for: m6A regulators as predictive biomarkers for chemotherapy benefit and potential therapeutic targets for overcoming chemotherapy resistance in small-cell lung cancer
Source: J Hematol Oncol. 2021 Nov 10;14:190. doi: 10.1186/s13045-021-01173-4 (PMC8579518; doi:10.1186/s13045-021-01173-4)
Supplement: Supplementary file 1 — Additional file 1. Supplementary Tables. [file 13045_2021_1173_MOESM1_ESM.docx]

**Additional file 1**

**Table S1.** The details of the 30 m^6^A regulators enrolled in this study.

| No. | Category | Gene ID | Official symbol | Description |
| --- | --- | --- | --- | --- |
| 1 | Writer | 56339 | METTL3 | Methyltransferase like 3 |
| 2 | Writer | 57721 | METTL14 | Methyltransferase like 14 |
| 3 | Writer | 79066 | METTL16 | Methyltransferase like 16 |
| 4 | Writer | 29081 | METTL5 | Methyltransferase like 5 |
| 5 | Writer | 9589 | WTAP | WT1 associated protein |
| 6 | Writer | 25962 | VIRMA | Vir like m6A methyltransferase associated |
| 7 | Writer | 64783 | RBM15 | RNA binding motif protein 15 |
| 8 | Writer | 29890 | RBM15B | RNA binding motif protein 15B |
| 9 | Writer | 23091 | ZC3H13 | Zinc finger CCCH-type containing 13 |
| 10 | Writer | 79872 | CBLL1 | Cbl proto-oncogene like 1 |
| 11 | Writer | 29063 | ZCCHC4 | Zinc finger CCHC-type containing 4 |
| 12 | Reader | 54915 | YTHDF1 | YTH N6-methyladenosine RNA binding protein 1 |
| 13 | Reader | 51441 | YTHDF2 | YTH N6-methyladenosine RNA binding protein 2 |
| 14 | Reader | 253943 | YTHDF3 | YTH N6-methyladenosine RNA binding protein 3 |
| 15 | Reader | 91746 | YTHDC1 | YTH domain containing 1 |
| 16 | Reader | 64848 | YTHDC2 | YTH domain containing 2 |
| 17 | Reader | 3181 | HNRNPA2B1 | Heterogeneous nuclear ribonucleoprotein A2/B1 |
| 18 | Reader | 3183 | HNRNPC | Heterogeneous nuclear ribonucleoprotein C |
| 19 | Reader | 2332 | FMR1 | FMRP translational regulator 1 |
| 20 | Reader | 8661 | EIF3A | Eukaryotic translation initiation factor 3 subunit A |
| 21 | Reader | 10642 | IGF2BP1 | Insulin like growth factor 2 mRNA binding protein 1 |
| 22 | Reader | 10644 | IGF2BP2 | Insulin like growth factor 2 mRNA binding protein 2 |
| 23 | Reader | 10643 | IGF2BP3 | Insulin like growth factor 2 mRNA binding protein 3 |
| 24 | Reader | 1994 | ELAVL1 | ELAV like RNA binding protein 1 |
| 25 | Reader | 10146 | G3BP1 | G3BP stress granule assembly factor 1 |
| 26 | Reader | 9908 | G3BP2 | G3BP stress granule assembly factor 2 |
| 27 | Reader | 7916 | PRRC2A | Proline rich coiled-coil 2A |
| 28 | Reader | 27316 | RBMX | RNA binding motif protein X-linked |
| 29 | Eraser | 79068 | FTO | FTO alpha-ketoglutarate dependent dioxygenase |
| 30 | Eraser | 54890 | ALKBH5 | AlkB homolog 5, RNA demethylase |

**Table S2**. The optimum cutoff survival analysis of 30 m^6^A regulators in the training cohort.

| M6A regulators | High expression (*N*) | Low expression (*N*) | *P* value | HR |
| --- | --- | --- | --- | --- |
| METTL3 | 38 | 12 | 0.206 | 0.611 |
| METTL14 | 22 | 28 | 0.008 | 2.588 |
| METTL16 | 7 | 43 | 0.043 | 0.250 |
| METTL5 | 26 | 24 | 0.017 | 2.441 |
| WTAP | 31 | 19 | 0.155 | 1.716 |
| VIRMA | 24 | 26 | 0.056 | 0.493 |
| RBM15 | 31 | 19 | 0.184 | 0.621 |
| RBM15B | 28 | 22 | 0.043 | 0.471 |
| ZC3H13 | 18 | 32 | 0.053 | 0.473 |
| CBLL1 | 29 | 21 | 0.218 | 1.583 |
| ZCCHC4 | 29 | 21 | 0.004 | 3.016 |
| FTO | 11 | 39 | 0.364 | 0.664 |
| ALKBH5 | 22 | 28 | 0.011 | 0.391 |
| YTHDF1 | 23 | 27 | 0.288 | 1.452 |
| YTHDF2 | 9 | 41 | 0.105 | 0.424 |
| YTHDF3 | 24 | 26 | 0.015 | 0.413 |
| YTHDC1 | 41 | 9 | 0.062 | 2.638 |
| YTHDC2 | 27 | 23 | 0.053 | 2.035 |
| HNRNPA2B1 | 8 | 42 | 0.008 | 3.071 |
| HNRNPC | 36 | 14 | 0.056 | 2.278 |
| FMR1 | 37 | 13 | 0.046 | 2.566 |
| EIF3A | 47 | 3 | 0.179 | 3.701 |
| IGF2BP1 | 26 | 24 | 0.026 | 0.455 |
| IGF2BP2 | 15 | 35 | 0.104 | 1.817 |
| IGF2BP3 | 18 | 32 | 0.005 | 0.299 |
| ELAVL1 | 16 | 34 | 0.197 | 0.588 |
| G3BP1 | 35 | 15 | 0.021 | 2.625 |
| G3BP2 | 37 | 13 | 0.015 | 3.434 |
| PRRC2A | 37 | 13 | 0.026 | 0.404 |
| RBMX | 36 | 14 | 0.033 | 2.584 |

**Table S3**. Clinical characteristics of the patients from multiple cohorts.

| Characteristics | Training Cohort  (*N*=50) | Validation Cohort (*N*=71) | Independent Cohort (*N*=79) |
| --- | --- | --- | --- |
| Age, year |  |  |  |
| <60 | 15 (30.00%) | 40 (56.43%) | 48 (60.76%) |
| ≥60 | 35 (70.00%) | 31 (43.66%) | 31 (39.24%) |
| Sex |  |  |  |
| Male | 34 (68.00%) | 54 (76.06%) | 60 (75.95%) |
| Female | 16 (32.00%) | 17 (23.94%) | 19 (24.05%) |
| Smoking history |  |  |  |
| Yes | 48 (96.00%) | 41 (57.75%) | 49 (62.03%) |
| No | 2 (4.00%) | 30 (42.25%) | 30 (37.97%) |
| SCLC staging |  |  |  |
| I | 17 (34.00%) | 23 (32.39%) | 16 (20.25%) |
| II | 10 (20.00%) | 25 (35.21%) | 32 (40.51%) |
| III | 15 (30.00%) | 23 (32.39%) | 31 (39.24%) |
| IV | 8 (16.00%) | 0 (0.00%) | 0 (0.00%) |
| OS state |  |  |  |
| Alive | 18 (36.00%) | 33 (46.48%) | 43 (54.43%) |
| Death | 32 (64.00%) | 38 (53.52%) | 36 (45.57%) |

SCLC, small cell lung cancer; OS, overall survival.

**Table S4**. Primer sequences used in the validation cohort for qPCR.

| Gene Name | Forward Primer | Reverse Primer |
| --- | --- | --- |
| G3BP1 | 5'-CACAAAGACCTCAGCGGGAT-3' | 5'-CTCACGGATTGGTCTGGGTC-3' |
| METTL5 | 5'-GTACGCGGAGTGGCAGAAA-3' | 5'-CAGAGGACGTTGCAGTAGC-3' |
| ALKBH5 | 5'-TCAAGCCTATTCGGGTGTCG-3' | 5'-ATCCACTGAGCACAGTCACG-3' |
| IGF2BP3 | 5'-TGCCACCATTCGGAACATCA-3' | 5'-AATCGACTTCTCAGCAGCCC-3' |
| RBMX | 5'-GACCGTGAAACCAACAAATCAA-3' | 5'-CATGTCTCTGGCTGCATCCTT-3' |
| YTHDF3 | 5'-GCTGGGTAGCTCCTCGTAAC-3' | 5'-CCAAAGTTTTCACTGCCCGC-3' |
| ZCCHC4 | 5'-CGGAGCTGTCAGTTCTTGGT-3' | 5'-AGCTCATGCAACCTTGGTGT-3' |
| GAPDH | 5'-AAATCAAGTGGGGCGATGCT-3' | 5'-CAAATGAGCCCCAGCCTTCT-3' |
