# Supplementary material for: m6A regulators as predictive biomarkers for chemotherapy benefit and potential therapeutic targets for overcoming chemotherapy resistance in small-cell lung cancer
Source: J Hematol Oncol. 2021 Nov 10;14:190. doi: 10.1186/s13045-021-01173-4 (PMC8579518; doi:10.1186/s13045-021-01173-4)

**Additional file 2**

**Figure S1** Kaplan–Meier survival analysis of small cell lung cancer grouped by the expression of m^6^A regulators in the training cohort, including ZCCHC4 (a) (High expression=29, Low expression=21), IGF2BP3 (b) (High expression=18, Low expression=32), METTL14 (c) (High expression=22, Low expression=28), HNRNPA2B1 (d) (High expression=8, Low expression=42), ALKBH5 (e) (High expression=22, Low expression=28), G3BP2 (f) (High expression=37, Low expression=13), YTHDF3 (g) (High expression=24, Low expression=26), METTL5 (h) (High expression=26, Low expression=24), G3BP1 (i) (High expression=35, Low expression=15), IGF2BP1 (j) (High expression=26, Low expression=24), PRRC2A (k) (High expression=37, Low expression=13), RBMX (l) (High expression=36, Low expression=14), METTL16 (m) (High expression=7, Low expression=43), RBM15B (n) (High expression=28, Low expression=22), FMR1 (o) (High expression=37, Low expression=13), YTHDC2 (p) (High expression=27, Low expression=23), ZC3H13 (q) (High expression=18, Low expression=32), HNRNPC (r) (High expression=36, Low expression=14), KIAA1429 (s) (High expression=24, Low expression=26), YTHDC1 (t) (High expression=41, Low expression=9), IGF2BP2 (u) (High expression=15, Low expression=35), YTHDF2 (v) (High expression=9, Low expression=41), WTAP (w) (High expression=31, Low expression=19), EIF3A (x) (High expression=47, Low expression=3), RBM15 (y) (High expression=31, Low expression=19), ELAVL1 (z) (High expression=16, Low expression=34), METTL3 (aa) (High expression=38, Low expression=12), YTHDF1 (ac) (High expression=23, Low expression=27), and FTO (ad) (High expression=11, Low expression=39).


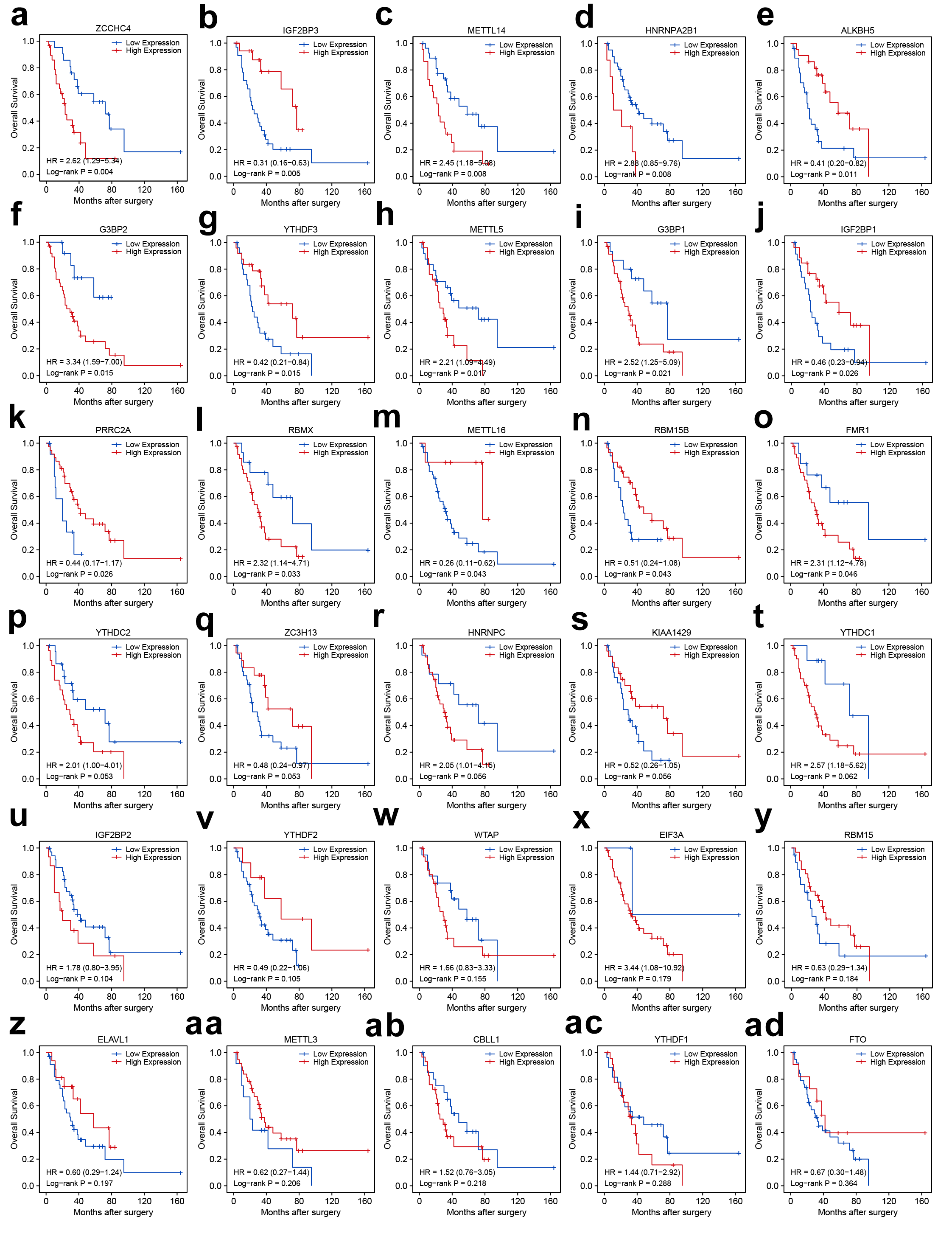


**Figure S2** Correlations between the expression of each significant m^6^A regulator in small cell lung cancer with chemotherapy from the training cohort.


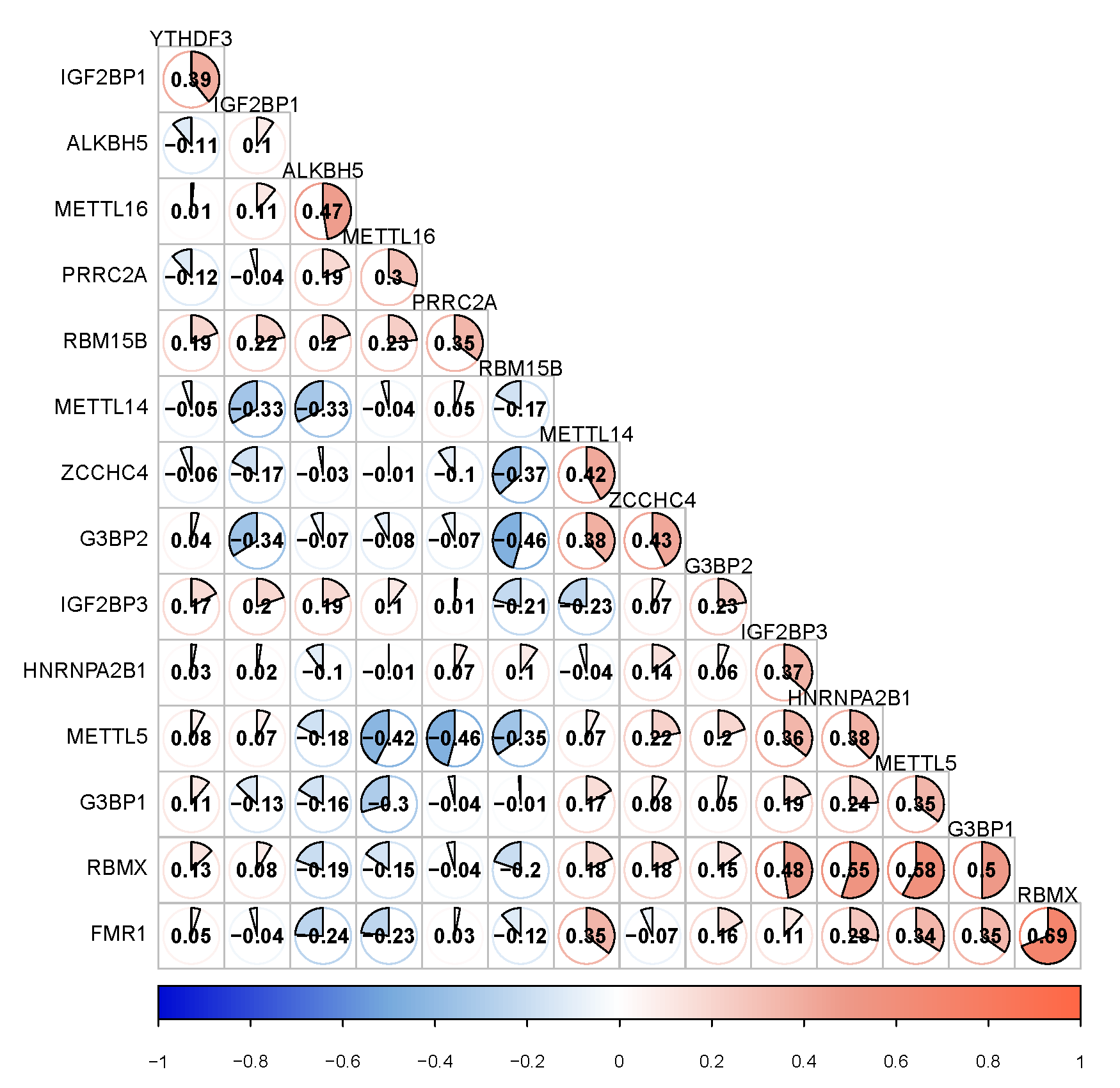


**Figure S3** Time-dependent ROC curves to evaluate predict accuracy of the m^6^A score and other clinicopathological parameters for overall survival at 1-,3- and 5-years in the training cohort (a). C-index of the m^6^A score and other clinicopathological parameters in the training cohort (b).


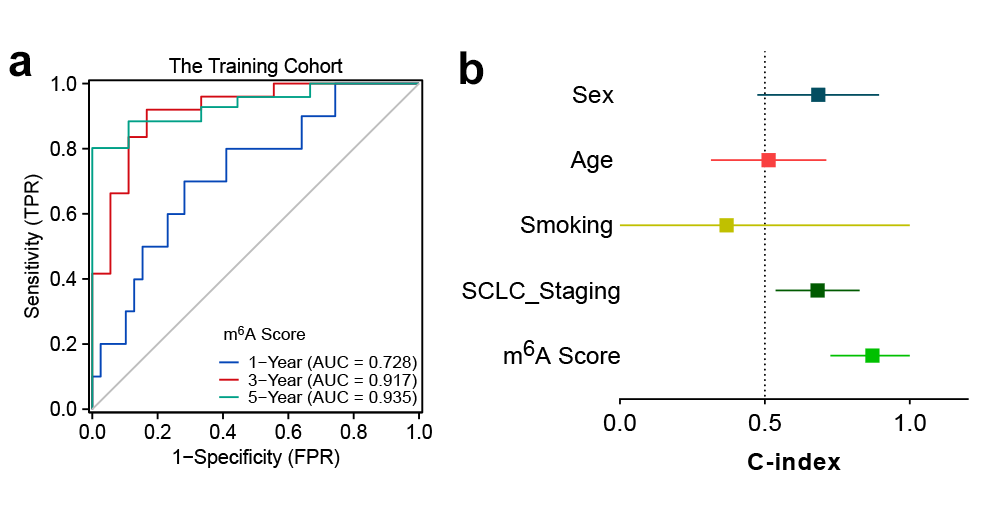


**Figure S4** Time-dependent ROC curves to evaluate predict accuracy of the m^6^A score and other clinicopathological parameters for overall survival at 1-,3- and 5-years in the validation cohort (a). Time-dependent ROC curves to evaluate predict accuracy of the m^6^A score and other clinicopathological parameters for relapse-free survival at 1-,3- and 5-years in the validation cohort (b). C-index of the m^6^A score and other clinicopathological parameters for estimating the overall survival in validation cohort (c). Time-dependent ROC curves compare the predict accuracy of the m^6^A score with other clinicopathological parameters for relapse-free survival at 5 years in the validation cohort (d). C-index of the m^6^A score and other clinicopathological parameters for estimating the relapse-free survival in validation cohort (e).


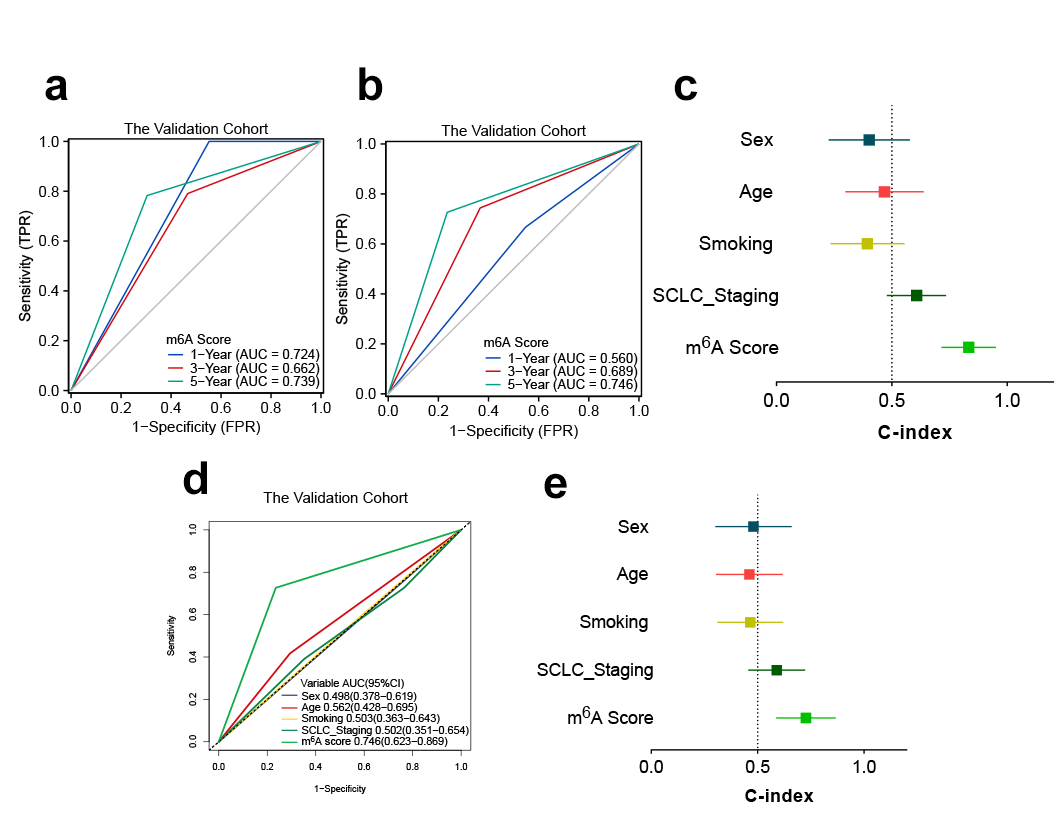


**Figure S5** Time-dependent ROC curves to evaluate predict accuracy of the m^6^A score and other clinicopathological parameters for overall survival at 1-,3- and 5-years in the independent cohort (a). Time-dependent ROC curves to evaluate predict accuracy of the m^6^A score and other clinicopathological parameters for relapse-free survival at 1-,3- and 5-years in the independent cohort (b). C-index of the m^6^A score and other clinicopathological parameters for estimating the overall survival in independent cohort (c). Time-dependent ROC curves compare the predict accuracy of the m^6^A score with other clinicopathological parameters for relapse-free survival at 5 years in the independent cohort (d). C-index of the m^6^A score and other clinicopathological parameters for estimating the relapse-free survival in independent cohort (e).


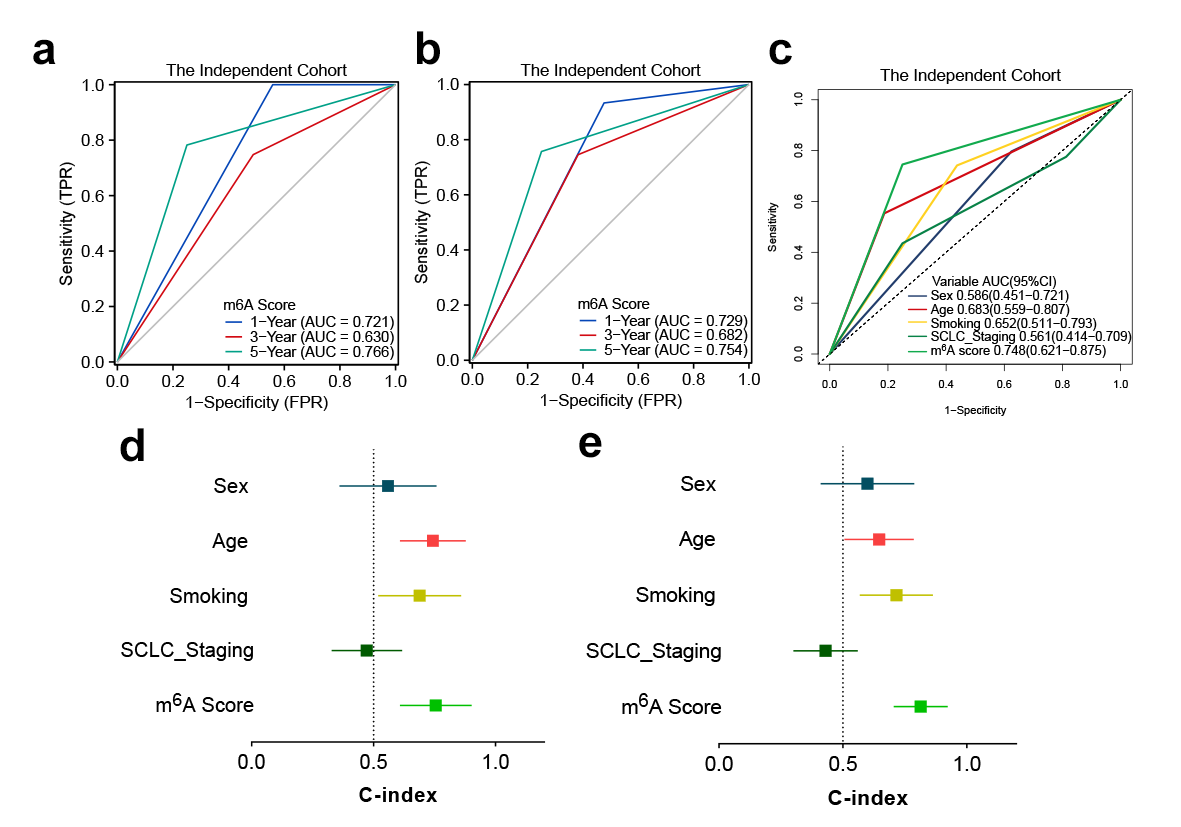


**Figure S6** Univariate Cox regression analysis of clinicopathological factors and the m^6^A score for relapse-free survival in patients from two different cohorts (a). Multivariate Cox regression analysis of clinicopathological factors and the m^6^A score for relapse-free survival in patients from two different cohorts (b).


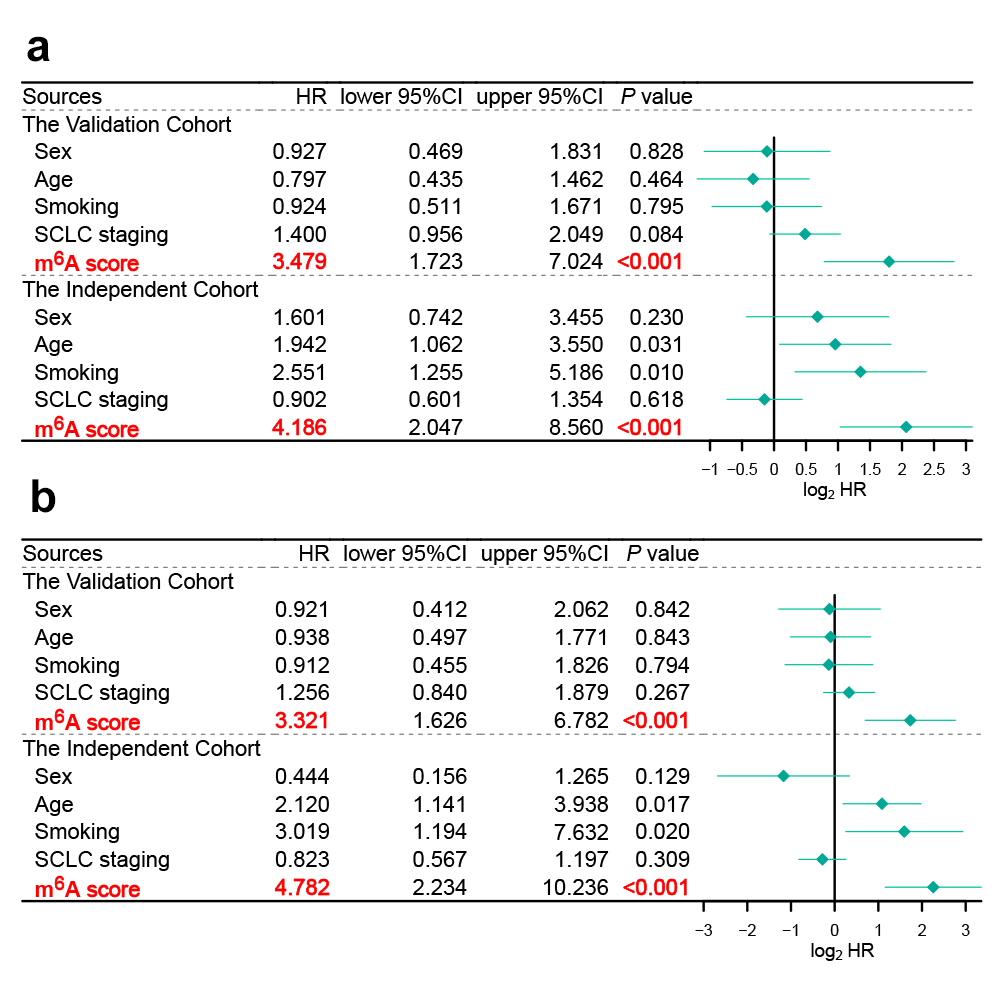


**Figure S7** qPCR confirmed the knockdown (KD) efficiency of *ZCCHC4* (a), *METTL5* (a), *G3BP1* (b), and *RBMX* (b) in the *in vitro* experiments.


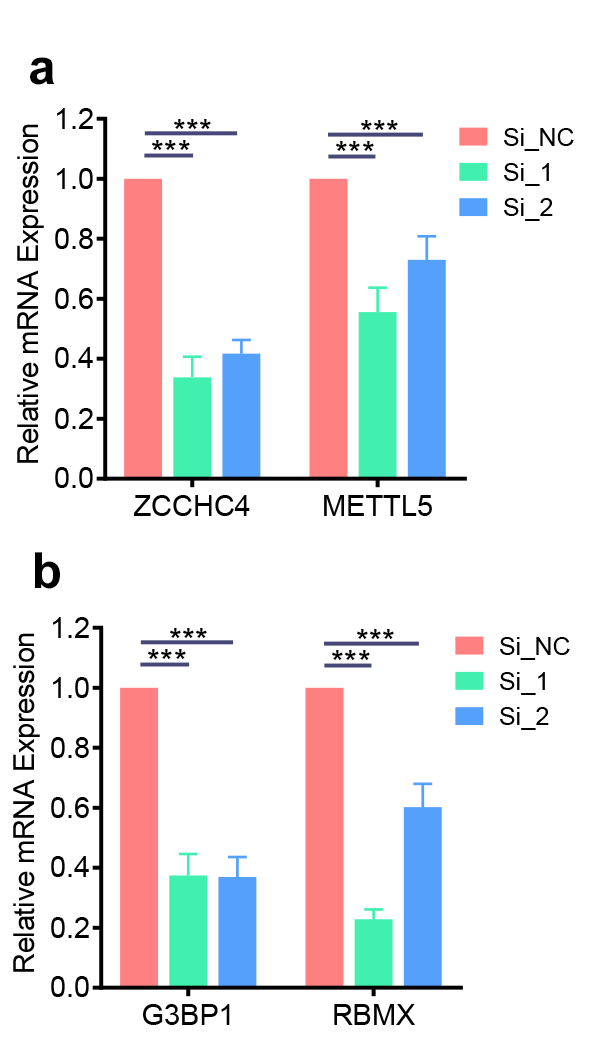

Supplement: Supplementary file 2 — Additional file 2. Supplementary Figures. [file 13045_2021_1173_MOESM2_ESM.docx]
