# Supplementary material for: m6A regulators as predictive biomarkers for chemotherapy benefit and potential therapeutic targets for overcoming chemotherapy resistance in small-cell lung cancer
Source: J Hematol Oncol. 2021 Nov 10;14:190. doi: 10.1186/s13045-021-01173-4 (PMC8579518; doi:10.1186/s13045-021-01173-4)
Supplement: Supplementary file 3 — Additional file 3. Supplementary materials and methods. [file 13045_2021_1173_MOESM3_ESM.docx]

**Additional file 3**

**Supplementary materials and methods**

**Collection of m^6^A regulators**

Totally, 30 m^6^A regulators—including eleven writers (METTL3, METTL14, METTL16, METTL5, WTAP, VIRMA, RBM15, RBM15B, ZC3H13, CBLL1, and ZCCHC4), seventeen readers (YTHDF1, YTHDF2, YTHDF3, YTHDC1, YTHDC2, HNRNPA2B1, HNRNPC, FMR1, EIF3A, IGF2BP1, IGF2BP2, IGF2BP3, ELAVL1, G3BP1, G3BP2, PRRC2A, and RBMX), and two erasers (FTO and ALKBH5)—were collected from several recently published studies ^1, 2, 3, 4^.

**Publicly available** **transcriptomic data of m^6^A regulators in small cell lung cancer (SCLC)**

The training cohort ^5^ were derived from Cbioportal (https://www.cbioportal.org/study/summary?id=sclc_ucologne_2015) and the clinicopathological features of these patients are summarized in Table S3. For analyzing the expression detail of *ZCCHC4*, *METTL5*, *G3BP1*, and *RBMX* in SCLCs and normal lung tissues, GSE40275 ^6^ from Gene Expression Omnibus (GEO) dataset (https://www.ncbi.nlm.nih.gov/geo/) were selected. All the transcriptomic data were first log2 transformed and the mean values were chosen as the final expression value of regulators with more than one probe.

**Patients and tissue specimens**

The implement process of this research was according to the ethical guidelines of the Declaration of Helsinki. The protocol was also approved by the Ethics Committee of our institute. All the patient’s data were analyzed in an anonymous form. Due to the retrospective nature of this study, the Ethics Committee waived the requirement of informed consent.

Totally, 150 cases of SCLC with ACT were collected as the validation cohort (N=71) and independent cohort (N=79). All these patients were diagnosed as SCLC in our hospital (the National Cancer Center) from January 2009 to October 2018. All the features of these patients are displayed in Table S3. No patients received neoadjuvant therapy before surgery. All the cases were pathologically reconfirmed by two pathologists. The day from surgery to recurrence, metastasis, or last follow-up was defined as relapse-free survival. And, the day from surgery to death was defined as the overall survival.

**RNA isolation and qPCR**

Only tissue biopsies certified with at least 70% tumor cells were collected for RNA insolation. We used ~30 μm sections from the FFPE samples and conducted the RNA extraction through using the Ambion RecoverAll Total Nucleic Acid Isolation Kit for FFPE (ThermoFisher, Waltham, MA, USA). All the operations are followed with the manufacturer’s instructions. After RNA quality control (A260/A280 ≥1.8), FastKing RT Kit（With gDNase）(KR116) was introduced to reverse transcribe the cDNA according to the manufacturer’s instructions (Tiangen, Beijing, China). Then, qPCR was carried out with SYBR Green (#208054, Qiagen, Dusseldorf, Germany). We chose the housekeeping gene—GAPDHA—as the internal control. The relative expression of the selected 7 regulators was calculated using the 2^−ΔΔCt^ method. The primers used in this study were summarized in supplementary Table S5.

**Tissue microarrays and** **immunohistochemistry staining**

Tissue microarrays (TMA) were consisted of 1.0 mm core biopsies per SCLCs from the most representative tumor area. Then, immunohistochemistry (IHC) was performed using the TMA according to standard protocols. Briefly, TMAs were incubated with primary antibodies—anti-ZCCHC4 (1:100 dilution, Abcam, ab154002), anti-IGF2BP3 (1:200 dilution, Abcam, ab273131), anti-ALKBH5 (1:2000 dilution, Abcam, ab195377), anti-YTHDF3 (1:200 dilution, Abcam, ab220161), anti-METTL5 (1:300 dilution, Novus, NBP1-56640), anti-G3BP1 (1:200 dilution, Abcam, ab181150), or RBMX (1:500 dilution, Abcam, ab190352) —followed by incubation with secondary antibodies. The staining of these m^6^A regulators was evaluated by a histologic score (H-score) system ^7^ through digital pathology image analysis. The value of H-score for each sample was ranged from 0 (no staining) to 300 (maximum immunoreactivity). Before the H-score was calculated, the intensity of immunostaining of each regulator was assessed as 0–3: 0, negative staining; 1, light staining; 2, moderate staining; 3, strong staining. Then, the H-score was conducted by a formula, H-score= 3 × (% at 3) + 2 × (% at 2) + 1 × (% at 1).

**RNA interference**

Small cell lung cancer cell line (NCIH446) was selected for ZCCHC4, METTL5, G3BP1, and RBMX knockdown. The commercially available siRNAs were brought from Syngentech Company (Beijing, China). When transfecting the cancer cells, the siRNAs were first mixed with Lipofectamine 3000 (Invitrogen, Carlsbad, CA, USA), and added to the cell culture medium according to the manufacturer’s instructions.

***In vitro* drug sensitivity assay**

After obtaining the half-maximal inhibitory concentrations (IC^50^) value for NCIH446, the cells (1 × 10^4^) from control or knockdown groups were seeded in 96-well plates and treated with cisplatin (10 μM) for 24 hours. Then Cell Counting Kit-8 (CCK-8; Dojindo, Japan) and plate clone formation assay were used to detect cell viability according to the manufacturer’s instructions.

**m^6^A score classifier construction and statistical analysis**

To clearly the m^6^A regulators that could potentially predict chemotherapy response, optimum cutoff survival analysis of these regulators in patients with chemotherapy from the training cohort using the “surv_cutpoint” function from the “survminer” R package ^8^ was carried out. Then, 15 regulators were filtered out, including ZCCHC4, IGF2BP3, METTL14, HNRNPA2B1, ALKBH5, G3BP2, YTHDF3, METTL5, G3BP1, IGF2BP1, PRRC2A, RBMX, METTL16, RBM15B, and FMR1, with the *P* values less than 0.05. Then, a popular method—the least absolute shrinkage and selection operator (LASSO) method with a Cox regression model ^9^—for variable selection was introduced to screen out the most useful candidates through the R package ‘‘glmnet‘‘. After that, seven regulators (*ZCCHC4*, *IGF2BP3*, *ALKBH5*, *YTHDF3*, *METTL5*, *G3BP1*, and *RBMX*) were identified, and we created the m^6^A score based on the individual expression of the 7 regulators, weighted by the regression coefficient: m^6^A score = (ZCCHC4 × 0.7942) + (IGF2BP3 × -0.2645) + (ALKBH5 × -0.4484) + (YTHDF3 × -0.6853) + (METTL5 × 0.4749) + (G3BP1 × 0.246) + (RBMX × 0.0911). To estimate the prognostic significance of the m^6^A score in multiple centers, Kaplan–Meier curve analysis with a two-tailed log-rank test using R package "survival" was applied. The R package "survival" was also used during the process of determining whether the m^6^A score is a significant independent prognostic factor across multiple cohorts. Time dependent ROC was realized by using the R package “timeROC”. When calculating the differences of m^6^A regulators between normal lung and SCLC samples, Mann–Whitney *U*-test was chosen. R software of version 3.5.1 (https://www.r-project.org) was used in this study for all the data processing and the image generation. A significant difference was considered only if the *P* value was less than 0.05 during all statistical process of this study.

**References**

1. Li, Y., et al., Molecular characterization and clinical relevance of m(6)A regulators across 33 cancer types*.* *Mol Cancer*. **18**:137 (2019).

2. Liu, J., Harada, B.T., and He, C., Regulation of Gene Expression by N(6)-methyladenosine in Cancer*.* *Trends Cell Biol*. **29**:487-499 (2019).

3. Huang, H., Weng, H., and Chen, J., m(6)A Modification in Coding and Non-coding RNAs: Roles and Therapeutic Implications in Cancer*.* *Cancer Cell*. **37**:270-288 (2020).

4. Nombela, P., Miguel-López, B., and Blanco, S., The role of m(6)A, m(5)C and Ψ RNA modifications in cancer: Novel therapeutic opportunities*.* *Mol Cancer*. **20**:18 (2021).

5. George, J., et al., Comprehensive genomic profiles of small cell lung cancer*.* *Nature*. **524**:47-53 (2015).

6. Kastner, S., et al., Expression of G protein-coupled receptor 19 in human lung cancer cells is triggered by entry into S-phase and supports G(2)-M cell-cycle progression*.* *Mol Cancer Res*. **10**:1343-58 (2012).

7. Yao, B., et al., PRMT1-mediated H4R3me2a recruits SMARCA4 to promote colorectal cancer progression by enhancing EGFR signaling*.* *Genome Med*. **13**:58 (2021).

8. Zhang, C., et al., Identification of a costimulatory molecule-based signature for predicting prognosis risk and immunotherapy response in patients with lung adenocarcinoma*.* *Oncoimmunology*. **9**:1824641 (2020).

9. Tibshirani, R., The lasso method for variable selection in the Cox model*.* *Stat Med*. **16**:385-95 (1997).
